# Association between time perspective and metacognition among Lebanese adults: the mediating role of mindfulness

**DOI:** 10.1186/s12888-023-05356-w

**Published:** 2023-12-05

**Authors:** Abdallah Chahine, Christian-Joseph El Zouki, Mariam Mhanna, Souheil Hallit, Sahar Obeid

**Affiliations:** 1 School of Medicine and Medical Sciences, Holy Spirit University of Kaslik, P.O. Box 446, Jounieh, Lebanon; 2 UFR de Médecine, Université de Picardie Jules Verne, 1 Rue des Louvels, Amiens, 80037 France; 3 Faculté d’Ingénierie et de Management de la Santé, 42 rue Ambroise Paré, 59120 LOOS, France; 4 Applied Science Research Center, Applied Science Private University, Amman, Jordan; 5 Research Department, Psychiatric Hospital of the Cross, Jal Eddib, Lebanon; 6 Social and Education Sciences Department, School of Arts and Sciences, Lebanese American University, Jbeil, Lebanon

**Keywords:** Time perspective, Metacognitions, Mindfulness, Lebanon

## Abstract

**Background:**

Mindfulness may serve as a component of metacognitive beliefs and can also be viewed as a form of time perspective. The interplay between time perspective and metacognitive beliefs remains understudied. Both aspects, however, display considerable stability over time and significantly influence an individual’s life and well-being. Lebanon, marked by its diverse and complex history, struggles with various political, social, and economic challenges. This study offers a valuable and unprecedented opportunity to examine these connections within a distinct cultural context, shedding light on the unique experiences of the Lebanese population. Therefore, our research aims to investigate the connection between time perspective and metacognition, with a focus on the role of mindfulness as a mediator.

**Methods:**

This cross-sectional study was conducted from August to November 2022 and involved participants from various regions of Lebanon. The questionnaire included sociodemographic data and scales such as the Arabic versions of the 15-item Short Form of the Zimbardo Time Perspective Inventory, the Metacognitions Questionnaire, the Freiburg Mindfulness Inventory and the Acceptance and Action Questionnaire (AAQ-2).

**Results:**

Our investigation recruited 423 participants. The analysis showed that individuals with a positive stance on their past, a hedonistic present, or a future-oriented outlook exhibited heightened levels of mindfulness. This elevated state of mindfulness, in turn, demonstrated a significant link to augmented cognitive self-consciousness (An increased introspection into one’s own thoughts). We also observed a direct association between a future-focused time perspective and high scores of cognitive self-consciousness. Furthermore, mindfulness emerged as a crucial mediator in the relationships between time perspectives and negative beliefs about the danger of worry. Similarly, individuals with a positive view on their past, a hedonistic present orientation, or a future-focused mindset demonstrated elevated levels of mindfulness, which was correlated with less negative beliefs about the danger of worry. Notably, a positive past perspective was directly associated with less negative views on worry and the subsequent loss of control, whereas higher future focused time perspective scores was significantly and directly associated with more negative beliefs about worry, whereas more future focused time perspective was significantly and directly associated with more negative beliefs about worry.

**Conclusion:**

Our findings found several meaningful associations between our variables, but it primarily underscored the significance of considering distinct subcomponents within mindfulness and psychopathological metacognition that may overlap, shedding light on their differential impacts on psychological well-being. We were also able to mirror the dual pathway theory of time perspective suggested in previous studies. These insights carry notable implications for the development and refinement of mindfulness-based and metacognitive interventions, emphasizing the need for tailored approaches that consider varying time perspectives. Continued investigation in this area promises to advance our understanding of these constructs and refine their practical applications in mental health interventions and well-being enhancement strategies.

## Background

Time, also known as the fourth dimension, is defined as the continuous and irreversible progress of events and existence, advancing indefinitely from the past to the present into the future [1]. This fundamental physical quantity has long been a crucial topic of study in Philosophy, Science, and Religion. Time constraints humans, and nearly all their actions and activities are based on this idea [2]. This building fabric of the universe influences our existence on a psychological and physical level, which explains the increased interest in recent years on its effect on behavioral psychology and its correlation with psychiatric occurrences such as depression and anxiety [3, 4]. Lately, several research showed possible implication of time perspective conceptualization with medical conditions and diseases [5, 6].

The time perspective theory posits that our understanding and experience of time affects our feelings, perceptions, and behavior. This notion is frequently viewed as an unconscious process that a person engages in to explain and make sense of the events in their life. According to the Zimbardo Time Perspective Theory, different perceptions of time exist between individuals and could be divided into five categories: Past Positive, Past Negative, Present Fatalistic, Present Hedonistic and Future. These time perspectives are thought to influence an individual thoughts, feelings, and behaviors, and can have an impact on various psychological outcomes [7–9]. The past-negative perspective is marked by negative memories and feelings of bitterness and remorse, this perspective has been related to the development and maintenance of psychopathology. The past-positive perspective is characterized by strong ties to family and a sentimental view of the past, as well as higher levels of well-being and life satisfaction. The present-hedonistic perspective prioritizes pleasure and often leads to risky behavior, addiction, and unhealthy lifestyles, while the present-fatalistic perspective involves feeling trapped in the present and a sense of helplessness. The future-focused perspective involves being goal-oriented and having a strong sense of urgency, but it may also come at the expense of close relationships and leisure time [7–9].

Zimbardo and Boyd stated that mindfulness is a holistic aspect of time perspective, and it has also been hypothesized that the way that we explore our memory of the past, experience the present and anticipate our future is a function of mindfulness. Being mindful means to be fully present in the current moment and to observe thoughts and emotions without judgment. Mindfulness is characterized as a state of non-elaborative, non-judgmental, present-focused awareness and involves an open, receptive approach to one’s experience, as well as a willingness to attend to and acknowledge one’s thoughts, feelings, and sensations without becoming overly reactive or overwhelmed by them [10–12]. Research has shown that mindfulness may be associated with a range of psychological outcomes, including improved mental health, emotion regulation, and well-being. Studies have also found that mindfulness practices may be effective in reducing symptoms of anxiety and depression, and may be helpful for individuals experiencing chronic pain, substance abuse, and other mental and physical health challenges [13–15]. Mindfulness is correlated with the five types of time perspectives. While Past Negative and both Present orientations would be negatively connected with mindfulness, Past Positive and Future would be favorably correlated with it [16–18]. An idea has been put forth that mindfulness can serve as a time perspective as well as an awareness of one’s own time perspective, creating a seemingly paradoxical relationship [17].

Jankowski and Holas [19] developed a comprehensive metacognitive model of mindfulness, and their conclusions suggest that both the neuropsychological evidence and theoretical conceptualizations of mindfulness point towards a metacognitive formulation of the phenomenon. Metacognition is a multifaceted cognitive process that allows individuals to be aware of and intentionally control their mental processes. It can be described as “thinking about thinking” and involves the mental structures and processes involved in the control and evaluation of cognition [20]. Research surrounding the self-regulatory executive function (S-REF) model specify that metacognition is important for optimal cognitive functioning and may be a factor in the development and maintenance of psychopathology. According to this model, dysfunctions in metacognitive control systems may contribute to unregulated thoughts and coping strategies associated with psychological disorders [21–23]. The Metacognition Questionnaire (MCQ-30) was based on the S-REF theory, and it consists of five metacognitive facets. The metacognitive beliefs “positive beliefs about worry,“ “negative beliefs about uncontrollability and danger of worry,“ “cognitive confidence,“ “cognitive self-consciousness,“ and “need to control thoughts” may be related to various psychological outcomes and disorders, such as anxiety, obsessive-compulsive disorder, and depression. “Positive beliefs about worry” refers to the belief that worrying is useful and necessary for problem-solving and decision-making. “Negative beliefs about uncontrollability and danger of worry” refers to the belief that one’s thoughts are uncontrollable and potentially harmful. “Cognitive confidence” is the belief that one’s thoughts and memories are reliable and accurate. “Cognitive self-consciousness” is the tendency to focus on and evaluate one’s own thoughts and mental processes. “Need to control thoughts” refers to the tendency to try to control or suppress unwanted thoughts and mental processes [24, 25]. Several studies pointed out the impact that those dysfunctional metacognitive beliefs can have on mental health [26–30]. There have been numerous attempts to develop a metacognitive model of mindfulness to explain how one affects the other, ranging from a basic mechanism put forth by Shapiro et al. [11] to a more comprehensive theory put out by Jankowski and Holas [19] that provides a more complex layer of knowledge. It was found that patients in a medical setting benefit from mindfulness’ promotion of metacognitive information processing [31]. Mindfulness was also found to decrease the manifestations of dysfunctional metacognitions, which in turn relieve the manifestations of psychiatric symptoms [32].

As already mentioned above, there might be a complex relationship between mindfulness, metacognition, and time perspective, particularly in the way that mindfulness is a component of metacognition, and that mindfulness can also be a form of time perspective and the act of being aware of one’s own time perspective. Furthermore, the relationship between time perspective and metacognitive beliefs has not been thoroughly examined in the literature. The dual pathway theory posits that an individual’s time perspective can directly or indirectly influence their well-being [33]. Additionally, it has been established that both time perspective and metacognitive beliefs exhibit a high degree of stability over time and have a significant impact on an individual’s life and well-being [7, 34]. Lebanon is a unique and diverse country that has faced numerous political, social, and economic challenges throughout the years. The country is still haunted by the memories of its golden era that was destroyed by its 15-year long civil war and its current dire state, which could have led to a fragmented perception of time among its population; the memories of the past, the unstable present, and the uncertain future are all intertwined in the nationwide discussion [35, 36]. The accumulation of crises have had a significant impact on the mental and emotional well-being of the Lebanese people [37–41]. While studies on variables such as metacognition and time perspective have been conducted in other countries [42, 43], there is a lack of research in the Lebanese context.

Uncovering the connections between our variables bears significant implications for practical applications, particularly in the realms of therapy and policy. This study’s insights into mental health dynamics can serve as a roadmap for tailored interventions, precisely targeting individuals with distinct time perspectives and metacognitive beliefs. This could ensure that treatment aligns with the unique needs of each individual. Additionally, these findings offer a blueprint for policy development, informing strategies aimed at bolstering the well-being of the population. From targeted mental health programs to comprehensive well-being initiatives, this research could provide tangible data for crafting procedures with a direct positive impact on individuals and communities alike. Therefore, we consider it crucial to investigate the mediating role of mindfulness in the relationship between time perspective and metacognitions among a sample of Lebanese adults.

We hypothesize that mindfulness would act as a mediator in the connection between past positive, present hedonism, future orientation, and metacognitive beliefs. In this scenario, elevated scores in time perspective would correspond to increased mindfulness and a reduction in metacognitive scores. Additionally, mindfulness may also play a mediating role in the link between past negative, present fatalistic, and metacognitive beliefs. Here, higher scores in time perspective would likely be associated with lower levels of mindfulness, consequently leading to higher metacognitive scores.

## Methods

### Study design and participants

This cross-sectional study was conducted from August to November 2022 and involved participants from various regions of Lebanon. Recruitment was done using respondent-driven and snowball sampling techniques. While no specific inclusion or exclusion criteria were defined for potential participants during the recruitment process, we requested that all respondents must be at least 18 years old and hold Lebanese citizenship, in conformity with the Ethics Committee’s green light. The study was conducted online using a Google Forms questionnaire and sent through social media sites and messaging applications. Participants were provided with an overview of the aims and general instructions of the study before enrolling. The use of convenience sampling methods may introduce bias, so efforts were made to include a diverse range of participants. There was no compensation offered for participation in the study.

### Minimal sample size calculation

We adopted the following formula proposed by Fritz and MacKinnon [44]: *n* = L/f^2 + k + 1. We computed it using an L of 7.85 for a 5% α error, a power of β = 80%, f = 0.14 for a small effect size, and k = 11 variables included in the model. Therefore, a minimum required sample size of 412 was considered for the current study.

### Study measures

The survey was conceived in Arabic, the official native language of Lebanon. The first section of the questionnaire included a description of the study’s objectives as well as other ethical considerations such as the assurance of respondents’ confidentiality and anonymity. A digital consent was also required, attesting to the participants’ willingness to voluntarily fill in the survey. The second segment of the survey consisted of questions that covered various socio-demographic variables, including age, sex, marital status, financial pressure, and education level. The household crowding index (HCI), which measures participants’ socioeconomic status (SES), was also considered. It was determined by dividing the number of inhabitants by the number of rooms in the home (excluding bathrooms and kitchens); a higher HCI score indicates a more crowded home, which is correlated with a lower SES [45]. Regarding their financial burden, respondents were asked to answer the question “How much pressure do you feel with regard to your personal financial situation in general?” on a scale from 1 to 10, with 10 referring to overwhelming pressure. The third part comprised the following measures:

The Short Form of the Zimbardo Time Perspective Inventory – Arabic Version (ZTPI-15-Ar). This 15-item self-report instrument yields time perspective scores within five distinct factors: Past Negative, Past Positive, Present Fatalistic, Present Hedonistic and Future Focused. Each statement is rated from “1” (Very uncharacteristic) to “5” (Very characteristic). Higher scores indicate a stronger propensity to employ a certain time perspective [7]. This version of the ZTPI was validated in a separate publication [46]. Some examples of items include “I think about the bad things that happened to me in the past”, “I enjoy stories about how things were in the good old days” and “Taking risks keeps my life from becoming boring”. In the Arabic validation study [46], McDonald’s ω values were as follows: past negative (ω = 0.84), past positive (ω = 0.66), present fatalistic (ω = 0.43), present hedonistic (ω = 0.61) and future focused (ω = 0.71) subscales.

The Metacognitions Questionnaire (MCQ-30). We used the Arabic version of this 30-item instrument [47]. This scale evaluates five degrees of metacognitive beliefs: “Positive beliefs about worry”, “Negative beliefs about uncontrollability and danger of worry”, “Cognitive confidence”, “Cognitive self-consciousness” and “Need to control thoughts”. Each subscale consists of six statements, each of which is rated on a Likert scale from 1 (Do not agree) to 4 (Strongly agree). Higher scores suggest higher maladaptive metacognitive beliefs [24]. The items in this scale are phrased as follows: “I cannot ignore my worrying thoughts”, “I think a lot about my thoughts” and “I have little confidence in my memory for places”. Cronbach’s alpha values in the original study were as follows: cognitive confidence (α = 0.93), positive beliefs (α = 0.92), cognitive self-consciousness (α = 0.92), negative beliefs (α = 0.91), and need to control thoughts (α = 0.72) subscales.

The Freiburg Mindfulness Inventory (FMI-Ar). The 14 items that make up the Arabic version of the Freiburg Mindfulness Inventory (FMI) cover several facets of mindfulness. Each item is graded on a 4-point Likert scale, with 1 denoting “Never” and 4 indicating “Always”. A higher overall score implies a greater experience of mindfulness [48]. The scale was previously translated into Arabic and validated in Lebanon [49]. Sample items within this scale are “I am open to the experience of the present moment”, “I accept unpleasant experiences” and “I watch my feelings without getting lost in them”. In the Arabic validation study [49], the McDonald’s ω and Cronbach’s α were excellent (= 0.92).

The Acceptance and Action Questionnaire (AAQ-2). This scale consists of seven items, each of which asks the individual to rate their level of agreement with a statement on a 7-point Likert scale, ranging from “never true” to “always true”. It assesses an individual’s psychological flexibility, which refers to the ability to be present in the moment and to engage in activities that align with personal values, even when faced with unpleasant thoughts, feelings, or experiences. This validated scale [50] was previously translated in Arabic [51]. Noteworthy example of items within this scale includes “Emotions cause problems in my life”, “Worries get in the way of my success” and “I’m afraid of my feelings”. To examine the mediating role of mindfulness in the relationship between time perspective and metacognition, we adjusted for psychological flexibility in our mediation models, therefore we isolated the effect of the mediator of interest (mindfulness) and made sure that the results you observe are not due to the influence of a very similar construct which is psychological flexibility. In the Arabic validation study [52], the Cronbach’s α value was 0.78.

### Statistical analysis

The SPSS software v.25 was used for the statistical analysis. No missing data was found in our database since all questions were required in the online survey. The metacognition subscales scores were considered normally distributed since the skewness and kurtosis values varied between − 1 and + 1. For the bivariate analysis, the Student t test was used to compare two means and the Pearson test was used to correlate two continuous variables. The mediation analysis was conducted using PROCESS MACRO v3.4, model 4, which computed 4 pathways: pathway A from the independent variable to the mediator, pathway B from the mediator to the dependent variable and pathway C (total effect) and C’ (direct effect) from the independent to the dependent variable reflecting the total and direct effects respectively. Results were adjusted over variables that showed a *p* < 0.25 in the bivariate analysis. *P* < 0.05 was deemed statistically significant.

## Results

McDonald’s ω values were modest in the total sample for the past negative (ω = 0.84), past positive (ω = 0.66), present fatalistic (ω = 0.43), present hedonistic (ω = 0.61) and future focused (ω = 0.71) subscales. They were very good for the cognitive confidence (ω = 0.87), positive beliefs (ω = 0.91), cognitive self-consciousness (ω = 0.87), negative beliefs (ω = 0.91), and need to control thoughts (ω = 0.80) subscales, mindfulness (ω = 0.86) and AAQ-2 (ω = 0.90) scales.

Our sample consisted of 423 participants, with a mean age of 29.19 ± 12.54 years and 68.6% females. Other characteristics of the sample can be found in Table 1.

**Table 1:** Sociodemographic and other characteristics of the participants (*N* = 423)

Variable	N (%)
**Sex**	
Male	133 (31.4%)
Female	290 (68.6%)
**Marital status**	
Single	315 (74.5%)
Married	108 (25.5%)
**Education**	
Secondary or less	22 (5.2%)
University	401 (94.8%)
	**Mean ± SD**
Age (in years)	29.19 ± 12.54
Financial pressure	4.95 ± 2.31
Household crowding index	0.98 ± 0.44
TP- past negative	10.00 ± 3.22
TP- past positive	11.70 ± 2.49
TP- present fatalistic	8.42 ± 2.53
TP- present hedonistic	9.24 ± 2.53
TP- future focused	12.01 ± 2.32
MCQ- cognitive confidence	13.09 ± 4.27
MCQ- positive beliefs	12.68 ± 4.74
MCQ- cognitive self-consciousness	19.13 ± 3.78
MCQ- negative beliefs	14.09 ± 5.35
MCQ- need to control thoughts	14.68 ± 4.14
Mindfulness	35.70 ± 7.32
Acceptance and Action score	22.89 ± 10.60

*TP *Time Perspective, *MCQ *Metacognition Questionnaire

### Bivariate analysis

The results of the bivariate analysis are summarized in Tables 2 and 3. All MCQ subscales were significantly associated with higher past negative and present fatalistic time perspectives (except cognitive self-consciousness). Higher past positive (*r* = − 0.12; *p* = 0.017) and future focused (*r* = − 0.18; *p* < 0.001) time perspectives was significantly correlated with less “lack of cognitive confidence”. Higher present hedonistic time perspective (*r* = 0.12; *p* = 0.016) was significantly correlated with more positive beliefs about worry. Higher mindfulness was significantly correlated with less lack of cognitive confidence (*r* = − 0.11; *p* = 0.020), negative beliefs about the uncontrollability of danger and worry (*r* = − 0.32; *p* < 0.001), and more cognitive self-consciousness (*r* = 0.15; *p* = 0.002). Finally, higher AAQ scores were significantly associated with higher MCQ subscales scores. A higher mean negative belief about uncontrollability of thoughts and worry was significantly found in single participants compared to married ones *t*(421) = 2.20, *p* = 0.028, whereas a higher mean of lack of cognitive confidence *t*(421) = 2.32, *p* = 0.021, and negative thought control *t*(421) = 2.45, *p* = 0.015 score was found in those with a secondary or less level of education compared to a university level.

**Table 2:** Bivariate analysis of factors associated with MCQ subscales scores

	1	2	3	4	5	6	7	8	9	10	11	12	13	14	15
1. Lack of cognitive confidence	1														
2. Positive beliefs about worry	0.15**	1													
3. Cognitive self-consciousness	− 0.01	0.10*	1												
4. Negative beliefs about the uncontrollability and danger of thinking and worry	0.28***	0.26***	0.27***	1											
5. Need to control thoughts	0.22***	0.26***	0.24***	0.60***	1										
6. Past negative	0.14**	0.15**	0.06	0.44***	0.26***	1									
7. Past positive	− 0.12*	0.02	0.09	− 0.07	− 0.04	− 0.04	1								
8. Present fatalistic	0.18***	0.17**	0.02	0.28***	0.17***	0.21***	0.19***	1							
9. Present hedonistic	0.06	0.12*	− 0.04	0.08	0.09	0.02	0.07	0.17***	1						
10. Future focused	− 0.18***	− 0.05	0.17**	− 0.06	− 0.04	− 0.11*	0.21***	− 0.13**	− 0.05	1					
11. Mindfulness	− 0.11*	− 0.05	0.15**	− 0.32***	− 0.18***	− 0.23***	0.16**	− 0.11*	0.10*	0.27***	1				
12. AAQ	0.23***	0.17***	0.13**	0.65***	0.50***	0.56***	− 0.04	0.28***	0.12*	− 0.21***	− 0.39***	1			
13. Age	0.03	0.03	− 0.02	− 0.12*	− 0.04	− 0.10*	0.11*	0.07	− 0.15**	0.05	0.07	− 0.23***	1		
14. Household crowding index	0.05	− 0.001	− 0.07	0.05	0.08	0.12*	− 0.06	0.08	0.14**	− 0.02	− 0.04	0.10*	− 0.17**	1	
15. Financial pressure	− 0.07	0.05	− 0.08	− 0.18***	− 0.13**	− 0.07	0.02	− 0.07	− 0.05	0.10*	0.16**	− 0.23***	− 0.06	− 0.01	1

Numbers indicate Pearson correlation coefficients; *p< .05; **p <.01; ***p <.001

**Table 3:** Bivariate analysis of categorical variables associated with the MCQ subscales

	Lack of cognitive confidence	Positive beliefs about worry	Cognitive self-consciousness	Negative beliefs about the uncontrollability and danger of thinking and worry	Need to control thoughts
**Sex**					
Male	13.17 ± 4.13	13.34 ± 4.92	19.13 ± 3.74	13.66 ± 5.20	14.97 ± 3.94
Female	13.05 ± 4.34	12.37 ± 4.63	19.13 ± 3.80	14.28 ± 5.41	14.54 ± 4.23
*P*	0.800	0.051	1	0.271	0.324
**Marital status**					
Single	13.03 ± 4.24	12.53 ± 4.66	19.17 ± 3.71	14.42 ± 5.41	14.75 ± 4.13
Married	13.27 ± 4.37	13.10 ± 4.95	19.00 ± 4.00	13.11 ± 5.09	14.45 ± 4.18
*P*	0.610	0.280	0.685	**0.028**	0.518
**Education**					
Secondary or less	15.14 ± 3.51	12.86 ± 5.21	19.73 ± 3.78	15.18 ± 5.21	16.77 ± 3.85
University	12.98 ± 4.28	12.67 ± 4.72	19.10 ± 3.78	14.03 ± 5.36	14.56 ± 4.13
*P*	**0.021**	0.849	0.445	0.324	**0.015**

### Mediation analysis

**Fig. 1:**
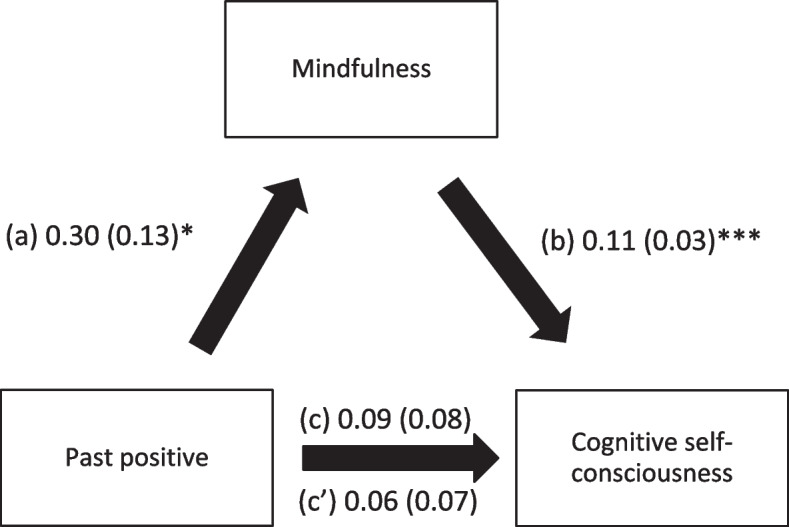
**a** Relation between past positive time perspective and mindfulness; (**b**) Relation between mindfulness and cognitive self-consciousness; (**c**) total effect of past positive time perspective on cognitive self-consciousness; (c’) Direct effect of past positive time perspective on cognitive self-consciousness. Numbers are displayed as regression coefficients (standard error). *p < 0.05; ***p < 0.001

**Fig. 2:**
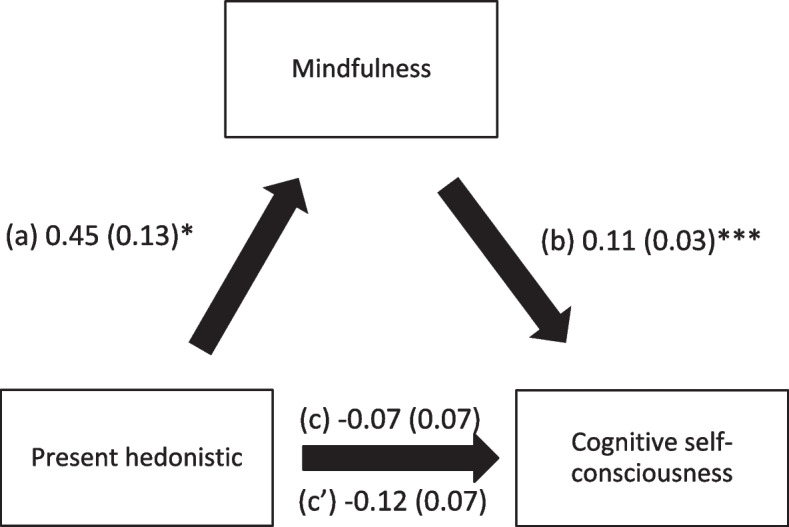
**a** Relation between present hedonistic time perspective and mindfulness; (**b**) Relation between mindfulness and cognitive self-consciousness; (**c**) total effect of present hedonistic time perspective on cognitive self-consciousness; (c’) Direct effect of present hedonistic time perspective on cognitive self-consciousness. Numbers are displayed as regression coefficients (standard error). *p < 0.05; ***p < 0.001

**Fig. 3:**
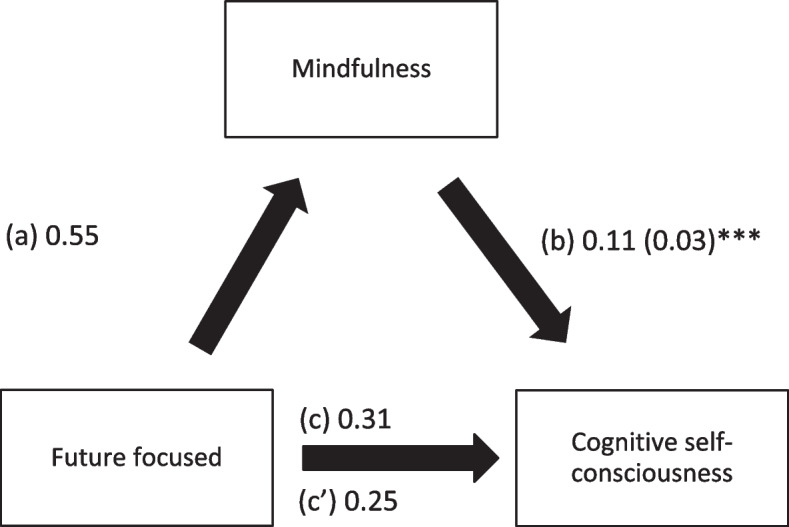
**a** Relation between future focused time perspective and mindfulness; (**b**) Relation between mindfulness and cognitive self-consciousness; (**c**) total effect of future focused time perspective on cognitive self-consciousness; (c’) Direct effect of future focused time perspective on cognitive self-consciousness. Numbers are displayed as regression coefficients (standard error). **p < 0.01; ***p < 0.001

Mindfulness mediated the associations between past positive, present hedonistic, future focused time perspective and cognitive self-consciousness. Higher scores of time perspective subscales were significantly associated with higher mindfulness, whereas higher mindfulness was significantly associated with higher cognitive self-consciousness. It is noteworthy that higher scores of future focused time perspective was the only one that showed a direct association with higher levels of cognitive self-consciousness (Table 4; Figs. 1, 2 and 3).

**Table 4:** Mediation analyses results, taking each time perspective subscale as an independent variable, mindfulness as the mediator and each MCQ subscale as dependent variables

Dependent variable	Direct effect			Indirect effect		
	Beta	SE	*P*	Beta	Boot SE	Boot CI
**Model 1: Lack of cognitive confidence as the dependent variable**						
Past negative	0.03	0.08	0.698	− 0.001	0.004	− 0.01; 0.01
Past positive	− 0.20	0.08	0.019	0.003	0.01	− 0.02; 0.03
Present fatalistic	0.21	0.09	0.016	− 0.001	0.01	− 0.01; 0.01
Present hedonistic	0.05	0.08	0.543	0.004	0.02	− 0.03; 0.03
Future focused	− 0.20	0.09	0.034	0.01	0.02	− 0.03; 0.05
**Model 2: Positive beliefs about worry as the dependent variable**						
Past negative	0.11	0.08	0.179	< 0.001	0.01	− 0.01; 0.01
Past positive	− 0.001	0.10	0.991	< 0.001	0.01	− 0.03; 0.03
Present fatalistic	0.21	0.10	0.033	< 0.001	0.01	− 0.01; 0.02
Present hedonistic	0.15	0.09	0.111	< 0.001	0.02	− 0.04; 0.03
Future focused	0.001	0.10	0.01; 0.99	< 0.001	0.02	− 0.05; 0.05
**Model 3: Linear regression taking cognitive self-consciousness as the dependent variable**						
Past negative	− 0.01	0.07	0.882	− 0.004	0.01	− 0.03; 0.02
Past positive	0.06	0.07	0.425	0.03	0.02	0.003; 0.07^a^
Present fatalistic	0.01	0.08	0.936	− 0.01	0.02	− 0.04; 0.03
Present hedonistic	− 0.12	0.07	0.100	0.05	0.02	0.02; 0.10^a^
Future focused	0.25	0.08	0.002	0.06	0.02	0.02; 0.11^a^
**Model 4: Negative beliefs about the uncontrollability and danger of thinking and worry as the dependent variable**						
Past negative	0.18	0.07	0.016	0.003	0.01	− 0.02; 0.02
Past positive	− 0.17	0.08	0.039	− 0.02	0.01	− 0.06; − 0.001^a^
Present fatalistic	0.28	0.08	0.001	0.01	0.01	− 0.02; 0.03
Present hedonistic	0.03	0.08	0.743	− 0.03	0.02	− 0.07; − 0.01^a^
Future focused	0.27	0.09	0.002	− 0.04	0.02	− 0.08; − 0.01^a^
**Model 5: Need to control thoughts as the dependent variable**						
Past negative	− 0.02	0.07	0.749	− 0.003	0.004	− 0.01; 0.01
Past positive	− 0.07	0.07	0.313	0.002	0.01	− 0.02; 0.02
Present fatalistic	0.06	0.08	0.430	− 0.001	0.01	− 0.01; 0.01
Present hedonistic	0.05	0.07	0.468	0.003	0.01	− 0.02; 0.03
Future focused	0.14	0.08	0.079	0.004	0.02	− 0.03; 0.04

^a^Indicates significant mediation

**Fig. 4:**
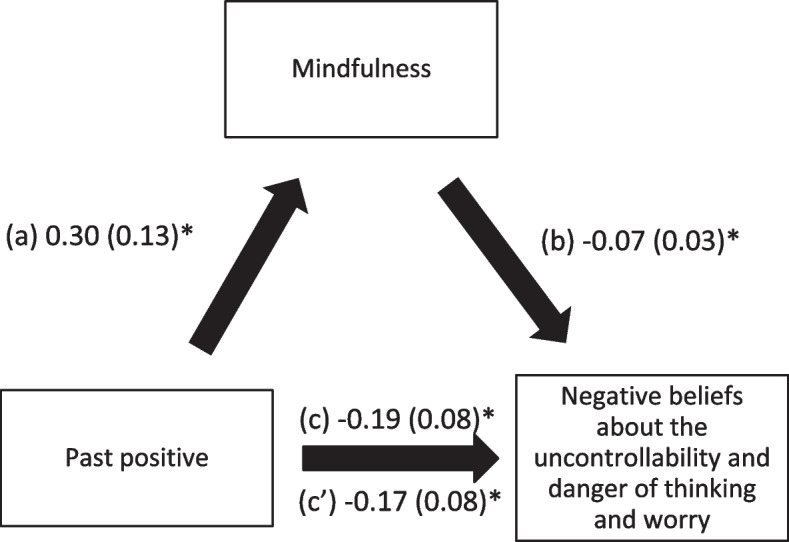
**a** Relation between past positive time perspective and mindfulness; (**b**) Relation between mindfulness and negative beliefs about the uncontrollability and danger of thinking and worry; (**c**) total effect of past positive time perspective on negative beliefs about the uncontrollability and danger of thinking and worry; (c’) Direct effect of past positive time perspective on negative beliefs about the uncontrollability and danger of thinking and worry. Numbers are displayed as regression coefficients (standard error). *p < 0.05

**Fig. 5:**
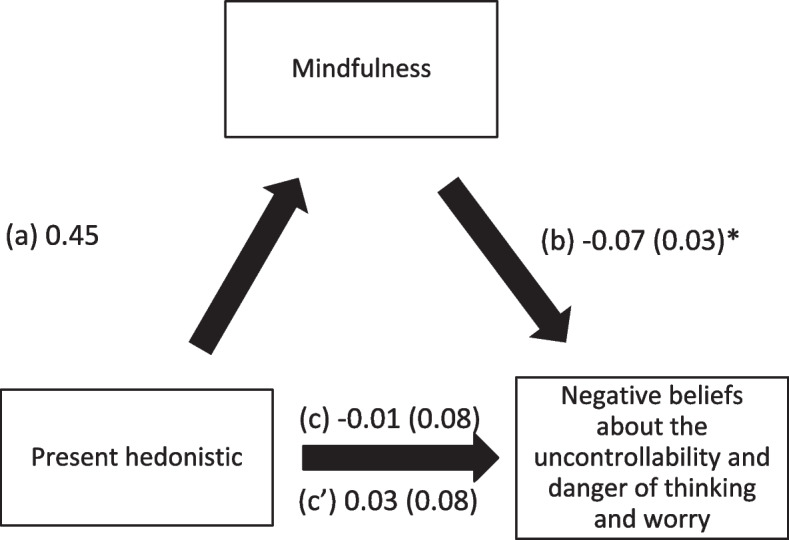
**a** Relation between present hedonistic time perspective and mindfulness; (**b**) Relation between mindfulness and negative beliefs about the uncontrollability and danger of thinking and worry; (**c**) total effect of present hedonistic time perspective on negative beliefs about the uncontrollability and danger of thinking and worry; (c’) Direct effect of present hedonistic time perspective on negative beliefs about the uncontrollability and danger of thinking and worry. Numbers are displayed as regression coefficients (standard error). *p < 0.05; ***p < 0.001

**Fig. 6:**
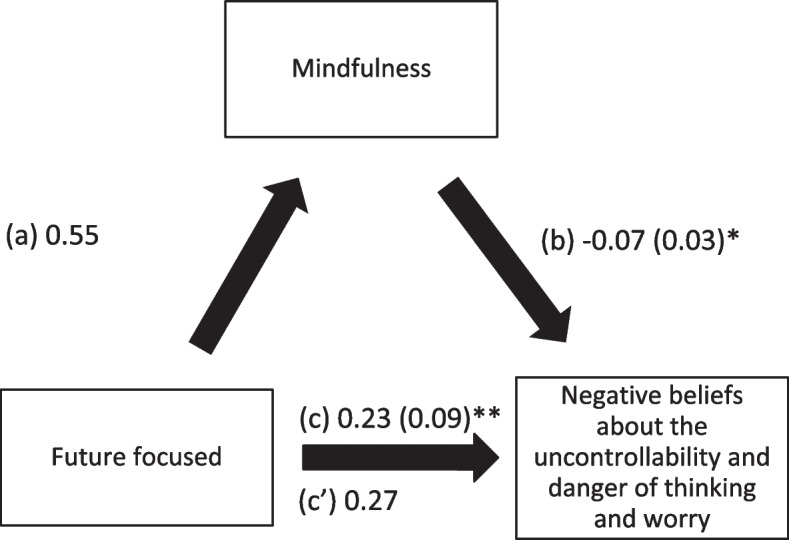
**a** Relation between future focused time perspective and mindfulness; (**b**) Relation between mindfulness and negative beliefs about the uncontrollability and danger of thinking and worry; (**c**) total effect of future focused time perspective on negative beliefs about the uncontrollability and danger of thinking and worry; (c’) Direct effect of future focused time perspective on negative beliefs about the uncontrollability and danger of thinking and worry. Numbers are displayed as regression coefficients (standard error). **p < 0.01; ***p < 0.001

Mindfulness also mediated the associations between past positive, present hedonistic, future focused time perspective and negative beliefs about the uncontrollability and danger of worry. Higher scores of the time perspective subscales were significantly associated with higher levels of mindfulness, whereas greater mindfulness was significantly associated with less negative beliefs about worry. Finally, higher past positive time perspective score was significantly and directly associated with less negative beliefs about worry, whereas more future focused time perspective was significantly and directly associated with more negative beliefs about worry. (Table 4; Figs. 4, 5 and 6).

## Discussion

The primary objective of this study was to investigate the relationship between time perspective, mindfulness, and pathological metacognitive processes, with a specific emphasis on the mediating role of mindfulness in this relationship. The analysis revealed several noteworthy findings, some of which were anticipated while others were surprising. It was observed that individuals who held a positive view of their past, prioritized hedonistic experiences in the present, or had a future-oriented mindset exhibited higher levels of mindfulness. This heightened state of mindfulness, in turn, displayed a significant connection to increased cognitive self-consciousness, indicating a greater introspective understanding of one’s own thoughts. Furthermore, there was a direct link observed between a future-focused perspective and heightened cognitive self-awareness. This finding may seem counterintuitive, as typically mindfulness would not be expected to be associated with a potentially problematic metacognitive process. Additionally, mindfulness emerged as a pivotal factor mediating the relationships between different time perspectives and negative beliefs regarding the dangers of worrying. Likewise, individuals with positive outlooks on their past, a focus on present pleasure, or a forward-looking mindset exhibited elevated levels of mindfulness, which was inversely related to reduced negative beliefs about the dangers and consequences of worrying. Notably, a positive view of the past was directly linked to holding less negative attitudes towards worrying and the subsequent feeling of losing control.

Previous studies found a significant correlation between a past positive orientation and mindfulness [17, 18]. Other research has suggested that mindfulness may be related to an increased ability to recall positive memories from the past [53]. Indeed, from a neuroscience perspective, an imbalance between top-down control by the cortex and bottom-up reactivity by subcortical regions, notably weak prefrontal control of the amygdala, may contribute to emotional biases in memory [54]. In response to unpleasant stimuli, there is increased amygdala activity and decreased anterior cingulate and prefrontal cortex activation in people who are depressed or anxious [55, 56]. This deficit in inhibitory regulation by the anterior cingulate and prefrontal results in an overactive amygdala as well as biases in memory towards negative stimuli and away from positive stimuli [57–60]. In fact, mindfulness and meditation practices have been shown to improve the prefrontal cortex’s ability to enhance its inhibitory functions, specifically on the amygdala [61–66], which may lead to an increased ability to recall positive events.

It does not come as a surprise that mindfulness and present hedonism are both very connected, as already demonstrated by previous studies. A person who has been taught to relish the moment could have acquired some expertise on how to consciously observe it. A person who has a hedonistic outlook on the present and seeks to enjoy it may also have a lot of attention to their internal processes, which may be related to the capacity for greater mindfulness [67]. Interestingly, a study conducted by Drake et al. in 2008, found that present hedonism has a negative relationship with mindfulness, which shows that there is a little tendency for people with low mindfulness to have a high present hedonistic score. This could be because individuals who score highly on hedonism tend to engage in dangerous endeavors and thrill-seeking behavior, which is thought to be an unmindful conduct [16]. This paradox may be the result of age and cultural differences, as was noted by previous studies [18, 67].

The positive association between a future focus and mindfulness is in harmony with some past studies [17, 18]. On the contrary, previous research by Drake et al. [16] and Vowinckel [67] found that there was no link between being focused on the future and mindfulness. The inconsistent findings in the literature could be accounted for by examining specific aspects of mindfulness, some of which were found to have a positive correlation with a future-oriented perspective while others were found to have a negative correlation. People who score high on a future scale tend to have an attitude that emphasizes achieving goals, completing tasks on time, and being responsible. It appears that this type of “responsible” attitude towards the future is associated with being aware of one’s actions and judging one’s inner experiences [67].

Future focus and present hedonism both have a very particular relationship with mindfulness. According to Seema Riin, and Anna Sircova [17], Present Hedonism and Future Focused are inherently opposite, especially regarding their relationship with mindfulness. In this Estonian study performed on a large sample of adult students, authors discussed that Present Hedonism was found to be inclined as a negative orientation towards time and negative attitudes, whereas Future Focused was inversely mirroring present hedonism. Therefore, it was concluded that “mindful” people have a more positive attitude toward the present and the future than people with a hedonistic approach to life. Additionally, unlike mindfulness, present hedonism is associated with constraining the time perspective to the present in a maladaptive manner. Consequently, it was found that mindfulness corresponds with the Future orientation of a person more than the present. Further studies are needed to understand the relationship between the abovementioned constructs considering possible confounding variables.

According to our mediation analysis, the three abovementioned Time Perspectives predict a higher level of mindfulness, which in turn was positively associated with cognitive self-consciousness. This may seem surprising because other research has suggested that cognitive self-consciousness, a dysfunctional metacognition, should be related to problematic symptoms [28], while mindfulness should be decreasing manifestations of psychiatric manifestations [68]. This could be due that an overemphasis or temporal bias towards one time perspective, that could lead to maladaptive strategies and negative outcomes [7]. However, mindfulness and cognitive self-consciousness both involve focusing inward, and these two variables were shown to share a common construct which is “observing”, or paying attention to thoughts, feelings, and behaviors [32]. Therefore, the relationship between these two variables may be due, in part, to this shared characteristic. Solem et al. [32] stipulated that experienced and novice meditators responded differently to stressors with a noticeable variability in how the brain was activated [69]. This underscores how proficiency in mindfulness could influence the connection between the act of “observing” and one’s overall well-being [70, 71]. The current research serves to fortify the idea that mindfulness may exhibit a relationship with the themes of metacognitive beliefs as outlined in the metacognitive theory propounded by Wells and Matthews [21, 22, 32, 72]. It has also been posited that the difficulties associated with deficient mindfulness, which may be correlated with some metacognitive processes, may stem from a lack of self-regulation and an inclination towards rumination, rather than seeking alternative cognitive strategies that enhance the capacity for flexibility and control [73].

As far as we are aware, limited research has explored the connection between dysfunctional metacognition and time perspective. The abovementioned findings imply a mutually reinforcing relationship between time perspective and metacognition, wherein each can potentially serve as an outcome of the other through direct or indirect pathways. It stands to reason that having a focus on the future, present, or past may involve thinking about one’s own thoughts, emotions, and mental activity, which is by definition a metacognitive process. To be more specific, individuals exhibiting high inclination towards a particular time perspective may also possess an elevated capacity for introspection and cognitive self-awareness. For example, people with a focus on their future might engage in introspection by constantly considering how their actions and decisions will impact their plans. On the other hand, hedonists could be cognitively self-aware due to a heightened focus on pleasure and emotions, a constant evaluation and reevaluation of their actions and thoughts, which could lead to a state of hyper-awareness towards their cognitive processes. Excessive self-reflection and self-awareness are integral facets of dysfunctional metacognition [21, 74, 75], thus having a significant focus on a certain time perspective may aid in these processes. As a result, there’s a potential risk that individuals with a future-oriented, past-positive, or present-hedonistic time perspective may have increased cognitive self-consciousness.

In harmony with our results, research has shown that people who have higher levels of mindfulness, particularly within the facets of *non-judgment*, *non-reactivity*, and *act aware*, tend to have lower levels of negative beliefs about worry [76]. The findings align with Wells’ theory which suggested that when applied correctly and in line with the metacognitive model, mindfulness may be useful in altering patients’ negative beliefs about worry [77].

Our research showed that when a person places a higher importance on present hedonism, they tend to also have higher mindfulness. In consequence, higher mindfulness was linked to fewer negative beliefs about worry. Plus, when someone has a strong focus on the future, they also tend to have higher levels of mindfulness which in turn is correlated with fewer negative beliefs about the lack of control over danger. But surprisingly enough, a stronger focus on the future was directly linked to more negative beliefs about uncontrollability of danger. Hedonists frequently adopt a live-in-the-moment philosophy and are happier than other people [33], but it’s thought-provoking to say that whether this outlook on the present turns out to be adaptive or maladaptive, specially that they are at higher risk of depression [7]. In comparison, future-oriented people tend to feel a little bit less happy in life than other time perspectives, but they paradoxically suffer less anxiety and depression, are more optimistic, and have both more positive and less negative affect than less future-oriented individuals [78–81]. These incongruent findings imply that the Future time orientation, like the Present Hedonistic time perspective, operates on well-being most likely through two different routes described as the “Dual Pathway Theory” [33]. We could mirror our results to this theory that analyses the ways in which time perspective influences well-being: (1) a direct pathway, where time perspectives have a direct impact on well-being, and (2) an indirect pathway, where time perspectives influence behaviors that in turn could affect an individual’s well-being. This dual pathway study provides a possible explanation to our results, with the authors’ suggestion that the future orientation has the least straightforward relationship with subjective wellbeing, potentially even showing a curvilinear pattern. Our results not only complement the direct pathway, indicating that individuals heavily focused on the future may harbor heightened negative beliefs about worry, but also substantiate the indirect pathway. This is evident in the observed relationship: more future focus leads to increased mindfulness, subsequently influencing beliefs about worry. Consequently, one’s future time perspective shapes behaviors (engaging in mindfulness practices) which in turn impact life circumstances, specifically by lowering negative beliefs about worry.

Kazakina’s research on older adults revealed that having a positive perspective on the past is the major predictor in determining life satisfaction and positive affect [82]. This insight can be applied to our current findings, which suggest that past positive experiences have a direct effect on negative beliefs about worry and an indirect effect via mindfulness, while present hedonistic or future focused perceptions require a mediator to reduce negative beliefs about worry. It is important to conduct additional research in order to build on the current findings and provide additional insights into the mechanisms behind these relationships.

### Clinical implications

Our results found that mindfulness played a mediating role in the association between time perspective and metacognition. Therefore, the way in which we view time can greatly influence multiple aspects of our life on a psychological and physical level. Therefore, we suggest that using Time Perspective Therapy [83] to balance our perspective on time, might increase mindfulness and decrease negative thoughts, plus this process could also be done reversely with Metacognitive Therapy [84]. Advocating for the inclusion of reliable mindfulness programs in educational curricula at an early age can equip individuals with effective coping strategies and emotional regulation techniques. Indeed, these findings suggest that promoting genuine forms of mindfulness through interventions and methodology that focus on a balanced time perspective may be beneficial in reducing some negative metacognitive beliefs and promoting wellbeing in individuals with worry-related issues. This may be useful in the management of several mental health conditions such as anxiety and depression [84, 85], and it could be crucial in addressing other issues related to negative metacognitive beliefs, such as OCD [84, 86, 87]. Moreover, public health campaigns to raise awareness about the impact of time perspective on mental health are needed given that this construct may not be widely recognized among the general public. This is specifically beneficial in the critical situation in Lebanon, particularly among vulnerable groups.

### Limitations

The present study has a few limitations that should be taken into consideration when interpreting the findings. One limitation is that the sample may not be representative of the general population due to potential selection bias, as some individuals may have declined to participate in the survey. Additionally, the distribution of sociodemographic characteristics was uneven, with women being overrepresented and the sample being relatively young. This may affect the generalizability of the results. Another limitation is that the responses were self-reported and were not evaluated by a health professional, which may result in information bias. The cross-sectional design of the study does not allow for the establishment of causality or temporality between the variables being studied. Future studies could work on longitudinal designs to provide a more in-depth understanding between these constructs over time. In addition, the use of self-report measures may be subject to social desirability bias, as participants may be inclined to present themselves in a more favorable light. The study may be limited by the specific measures used to assess the constructs of interest, as these measures may not capture the full range of individual differences in metacognition, time perspective, and mindfulness. The McDonald’s omega of the “fatalistic” subscale suggests a relatively weak level of internal consistency. This implies that the items within this specific subscale may not be effectively measuring a singular and coherent construct; consequently, results should be interpreted with caution. One further limitation to consider is the potential for participant recall bias, as the study relies on participants’ memories and recollections of their experiences. Furthermore, the use of mindfulness as a single mediator to explain the relationship between metacognition and time perspective may oversimplify the complexity of this relationship. It would be beneficial to examine the various components of mindfulness separately, as there may be some elements that have both positive and negative associations with certain variables, rather than considering mindfulness as a single entity. It is important to recognize these limitations and to consider conducting further research to address them.

## Conclusion

This pioneering study is one of the first to explore the mediating role of mindfulness in the association between time perspective and metacognition. Additionally, we can consider this research as new addition to the field as it is among the first to examine the intersection of time perspective and metacognition, specially within the cultural context of Lebanon. We found that mindfulness mediated the associations between past positive, present hedonistic and future focused time perspective with metacognitive beliefs such as cognitive self-consciousness and negative beliefs about uncontrollability of danger and worry. Expanding the research to include diverse cultural contexts to investigate how cultural differences may influence the relationship between time perspective, metacognition, and mindfulness could provide valuable insights into the universality or context-specific nature of these associations. Additional research is necessary to fully dissect the relationship between mindfulness and metacognition (cognitive self-consciousness and negative beliefs about worry), knowing that these two concepts may possess similar subcomponents. Given that mindfulness and dysfunctional metacognitions have distinct effects on psychopathology and well-being, it is important to fully understand the relationship between these two constructs and to (1) accurately study and understand each one of these variables without the risk of misinterpretation due to overlap in their subcomponents and (2) effectively design and comprehend mindfulness techniques and procedures. We also suggest expanding our research’s objective by working on the newly introduced Future Negative and Future Positive subfactors of the Future factor of the Zimbardo Time Perspective Inventory [88]. Finally, future research could further analyze the relationship between metacognition, mindfulness and “Balanced Time Perspective”, given that the latter is a primary topic of attention and discussion [89].

## Data Availability

All data generated or analysed during this study are not publicly available due the restrictions by the ethics committee (data are owned by the Psychiatric Hospital of the Cross). The dataset supporting the conclusions is available upon request to Ms. Rana Nader (rnader@naderlawoffice.com), a member of the ethics committee at the Psychiatric Hospital of the Cross.
